# Protein associated with SMAD1 (PAWS1/FAM83G) is a substrate for type I bone morphogenetic protein receptors and modulates bone morphogenetic protein signalling

**DOI:** 10.1098/rsob.130210

**Published:** 2014-02-19

**Authors:** Janis Vogt, Kevin S. Dingwell, Lina Herhaus, Robert Gourlay, Thomas Macartney, David Campbell, James C. Smith, Gopal P. Sapkota

**Affiliations:** 1 MRC Protein Phosphorylation and Ubiquitylation Unit, University of Dundee, Dow St., Dundee DD1 5EH, UK; 2 Division of Systems Biology, MRC National Institute for Medical Research, The Ridgeway, Mill Hill, London NW7 1AA, UK

**Keywords:** bone morphogenetic protein, SMAD1, FAM83G, PAWS1, ALK3, BMPR1

## Abstract

Bone morphogenetic proteins (BMPs) control multiple cellular processes in embryos and adult tissues. BMPs signal through the activation of type I BMP receptor kinases, which then phosphorylate SMADs 1/5/8. In the canonical pathway, this triggers the association of these SMADs with SMAD4 and their translocation to the nucleus, where they regulate gene expression. BMPs can also signal independently of SMAD4, but this pathway is poorly understood. Here, we report the discovery and characterization of PAWS1/FAM83G as a novel SMAD1 interactor. PAWS1 forms a complex with SMAD1 in a SMAD4-independent manner, and BMP signalling induces the phosphorylation of PAWS1 through BMPR1A. The phosphorylation of PAWS1 in response to BMP is essential for activation of the SMAD4-independent BMP target genes *NEDD9* and *ASNS*. Our findings identify PAWS1 as the first non-SMAD substrate for type I BMP receptor kinases and as a novel player in the BMP pathway. We also demonstrate that PAWS1 regulates the expression of several non-BMP target genes, suggesting roles for PAWS1 beyond the BMP pathway.

## 2. Introduction

The bone morphogenetic proteins (BMPs) belong to the transforming growth factor β (TGF-β) family of ligands, and play key roles in development and tissue homeostasis [1–5]. BMPs control many cellular processes, including differentiation, proliferation, survival, migration and morphogenesis in diverse biological contexts [1], and as a result abnormal BMP signalling is associated with the pathogenesis of several human diseases, including bone and developmental defects as well as cancer [6–10]. The actions of BMP ligands on their target cells are tightly regulated. This is achieved through several processes, from limiting access of BMPs to their receptors by secreted molecules such as noggin, to the regulation of the activities of the downstream pathway components [11–14].

Upon binding their cognate receptor serine/threonine kinase pairs, BMP ligands facilitate the phosphorylation and activation of type I BMP receptors (ALKs 2, 3 and 6) by type II BMP receptors (BMPRII, ActRIIA and ActRIIB). The type I receptors, in turn, phosphorylate the highly conserved receptor-regulated SMAD transcription factors (R-SMADs 1, 5 and 8) on two serine residues at their conserved C-terminal tail SXS motifs. The phosphorylation of R-SMADs triggers their association with SMAD4 and their subsequent translocation to the nucleus, where SMAD transcriptional complexes assemble to regulate the expression of hundreds of target genes [14,15]. The SMAD4-dependent transcriptional programme driven by the BMP ligands is often referred to as ‘canonical’ BMP signalling.

Consistent with the central role that SMAD4 plays in BMP and TGF-β signalling, the loss of SMAD4 expression is a common feature in many human cancers [16,17]. However, many studies have suggested that BMP ligands can also drive SMAD4-independent and, in some cases, even SMAD1/5/8-independent signalling, collectively termed as ‘non-canonical’ BMP signalling [18–23]. For example, in SW480 colorectal cancer cells, which are SMAD4-deficient, BMPs modulate the transcription of about 90 genes, including *NEDD9*, *ASNS* and *PTEN* [18,23], and non-canonical signalling influences a range of cellular responses, including the suppression of cell proliferation and chemotaxis [19–21,23]. However, the mechanisms by which BMP activates non-canonical signalling remain elusive.

In the course of a proteomic approach aimed at uncovering novel regulators of the BMP pathway, we identified FAM83G (hereafter referred to as protein associated with SMAD 1; PAWS1) as a SMAD1 interactor. PAWS1 is conserved in vertebrates but no biochemical roles have yet been reported. PAWS1 belongs to a family of hypothetical proteins, FAM83A–H, defined by the presence of a conserved N-terminal domain of unknown function termed DUF1669, which contains a putative pseudo-phospholipase D motif [24]. Recently, FAM83A and B have been reported to be oncogenes and mediators of resistance to tyrosine kinase inhibitors [25,26]. Mutations in FAM83H have been implicated in amelogenesis imperfecta, a condition characterized by dental-enamel defects [27]. However, the precise biochemical roles of the FAM83 family of proteins remain undefined.

Here, we demonstrate that PAWS1 forms a macromolecular complex with SMAD1 that is independent of SMAD4. In addition, we show that PAWS1 is a novel substrate for ALK3 and that BMP-induced phosphorylation of PAWS1 regulates the expression of the SMAD4-independent BMP target genes *NEDD9* and *ASNS*. In the course of our experiments, we show that PAWS1 regulates the BMP pathway and that it can regulate the expression of several genes independent of BMP stimulation.

## 3. Results

### 3.1. PAWS1/FAM83G associates with SMAD1

**Figure 1.**
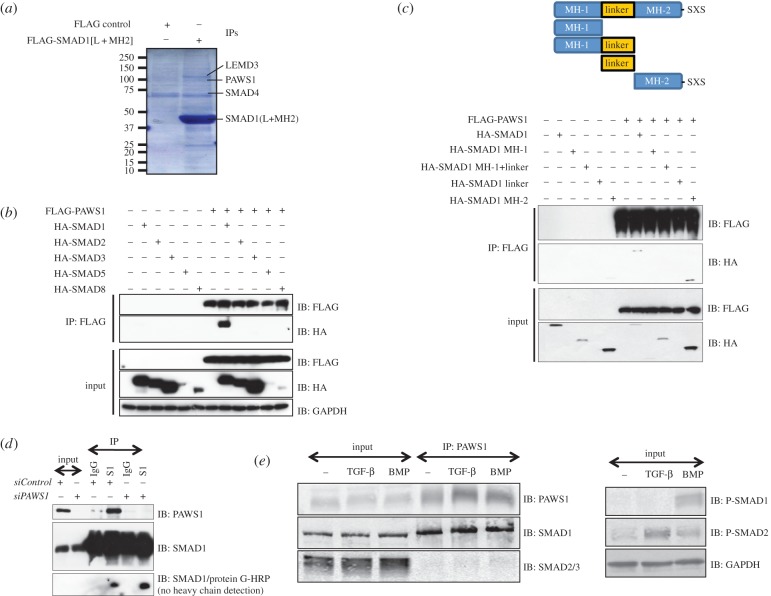
PAWS1 interacts with SMAD1. (*a*) Anti-FLAG IPs from HEK293 extracts transfected with vectors either encoding FLAG control or FLAG-tagged SMAD1[L + MH2] fragment were incubated with HeLa extracts. Elution was performed with 3X FLAG peptide. Eluted proteins were denatured and resolved by SDS–PAGE and the gel was Coomassie-stained. Gel pieces (2 mm) covering the entire lane of each sample were excised for identification by mass fingerprinting. The positions of some of the identified proteins are indicated. (*b*) HEK293 cells were transfected with the indicated HA-SMAD constructs either alone or together with FLAG-PAWS1 construct. The extracts and anti-FLAG IPs were analysed by immunoblotting using the indicated antibodies. (*c*) HEK293 cells transfected with constructs encoding either HA-SMAD1 or indicated HA-SMAD1 truncation mutants either individually or together with construct encoding FLAG-PAWS1. The extracts and anti-FLAG IPs were analysed by immunoblotting using the indicated antibodies. (*d*) HaCaT cells were transfected with a pool of two different *siRNA*s against either PAWS1 (150 pM each), or with *siRNA* against FOXO4, for 48 h prior to lysis. Extracts and IPs, using either anti-SMAD1 antibody or pre-immune IgG, were analysed by immunoblotting using the indicated antibodies. For SMAD1/protein-G-HRP immunoblot, the membrane was first blocked in 5% milk containing 500 ng ml^−1^ protein G, incubated with SMAD1 antibody as primary, and protein-G HRP was used as secondary. This strategy excludes the detection of antibody heavy chains in IP samples. (*e*) HaCaT cells were treated with either BMP-2 (25 ng ml^−1^; 1 h), TGF-β (50 pM; 1 h) or left untreated prior to lysis. Extracts and anti-PAWS1 IPs were analysed by immunoblotting with the indicated antibodies.

In an effort to uncover novel regulators of the BMP pathway, we used a proteomic approach to identify partners of SMAD1. An N-terminally FLAG-tagged SMAD1 fragment comprising the linker and MH2 (L + MH2) domains, or an empty vector control, were expressed in HEK293 cells which were then immunoprecipitated with anti-FLAG antibody. The immunoprecipitates (IPs) were incubated with cleared HeLa extracts, and interacting proteins were resolved by SDS–PAGE, excised, digested with trypsin and identified by mass fingerprinting (figure 1*a*). LEMD3, SMURF2 and SMAD4, previously reported to be interacting partners of SMAD1, were identified only in FLAG-SMAD1[L + MH2] IPs [28–30]. We also identified a previously uncharacterized protein termed FAM83G (figure 1*a*), which we renamed protein associated with SMAD1 (PAWS1).

To verify the interaction between PAWS1 and SMAD1 and to assess the specificity of their interaction, a mammalian expression construct encoding PAWS1 with a FLAG tag at the N-terminus (FLAG-PAWS1) was co-expressed in HEK293 cells with constructs encoding R-SMADs with N-terminal haemagglutinin (HA)-tags. HA-SMAD1 was identified in FLAG-PAWS1 IPs, whereas HA-SMADs 2 and 3 were not (figure 1*b*). The expression of HA-SMAD5 and HA-SMAD8 was too low to assess their interactions with FLAG-PAWS1 (figure 1*b*). To overcome this, FLAG-PAWS1 was co-expressed in HEK293 cells with constructs encoding SMADs 1, 5 and 8 containing N-terminal GFP tags (the electronic supplementary material, figure S1*a*). GFP-SMAD1, GFP-SMAD5 and GFP-SMAD8 were all identified in FLAG-PAWS1 IPs, suggesting that BMP-SMADs interact with PAWS1. GFP-SMAD4 did not interact with FLAG-PAWS1 (the electronic supplementary material, figure S1*b*).

To map the interaction between PAWS1 and SMAD1, FLAG-PAWS1 was co-expressed with N-terminal HA-tagged truncation fragments of SMAD1 in HEK293 cells (figure 1*c*). As expected, FLAG-PAWS1 interacted with full-length SMAD1 (figure 1*c*). Of the SMAD1 fragments, only the HA-MH2 domain of SMAD1 interacted with FLAG-PAWS1, whereas the HA-MH1 + linker domain did not interact (figure 1*c*). The expression of the HA-MH1 domain or the HA-linker domain was not detected. We also co-expressed N-terminal FLAG-tagged truncation fragments of SMAD1 in HEK293 cells with HA-PAWS1. HA-PAWS1 was detected in FLAG IPs of WT SMAD1, the MH1 domain and the MH2 domain, but not the linker (the electronic supplementary material, figure S1*c*).

To ask whether endogenous SMAD1 and PAWS1 interact, SMAD1 IPs from human keratinocyte HaCaT extracts were subjected to immunoblot analysis with an anti-PAWS1 antibody (figure 1*d*). Endogenous PAWS1 was detected in SMAD1 IPs but not in IPs using pre-immune IgG (figure 1*d*). SMAD1 IPs also failed to pull down PAWS1 from HaCaT cells transfected with PAWS1 *siRNA*, which resulted in almost complete loss of PAWS1 protein expression (figure 1*d*). Similarly, we detected endogenous SMAD1, but not SMAD2/3, in PAWS1 IPs from HaCaT cell extracts (figure 1*e*). Treatment of cells with BMP or TGF-β, to induce phosphorylation of SMAD1 and SMAD2/3, respectively, did not significantly alter the association of PAWS1 with SMAD1 or SMAD2/3 in extracts (figure 1*e*).

### 3.2. PAWS1 forms a complex with SMAD1 independent of SMAD4

**Figure 2.**
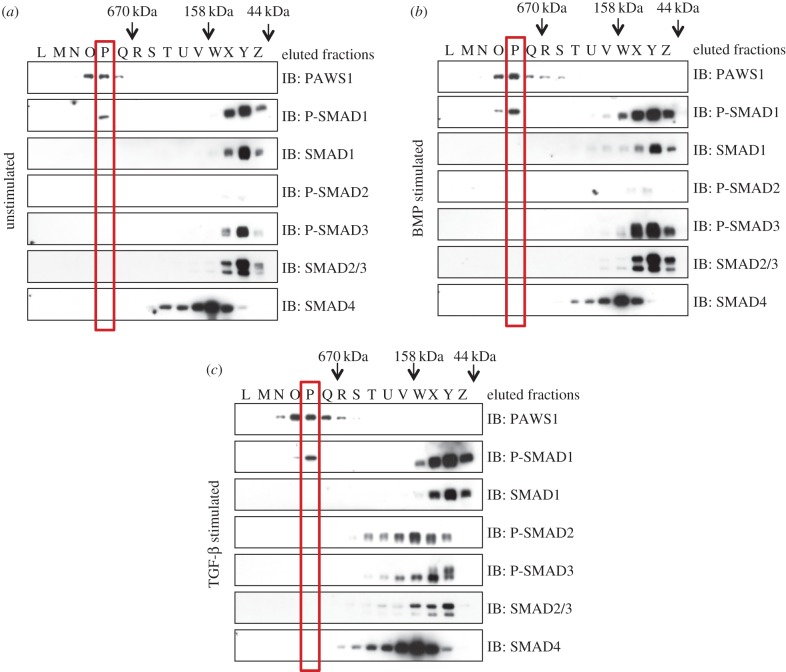
Size-exclusion chromatography. (*a*) Unstimulated HaCaT cell extracts were fractionated by gel filtration chromatography on a Superose 10/300 GL column (GE Healthcare). Five microlitres of each recovered fraction was resolved by SDS–PAGE and subsequently analysed by immunoblotting using the indicating antibodies. (*b*) Same as (*a*) except the cells were treated with BMP-2 (25 ng ml^−1^) for 1 h prior to lysis. (*c*) Same as (*a*) except the cells were treated with TGF-β (50 pM) for 1 h prior to lysis.

The observation that endogenous PAWS1 and SMAD1 interact with each other prompted us to ask whether they form a macromolecular complex. To this end, extracts from untreated HaCaT cells or from cells treated with BMP or TGF-β were separated into 32 fractions by size-exclusion chromatography (figure 2). Under basal unstimulated conditions, SMAD1 and SMAD2/3 were mostly detected in fractions corresponding to their predicted molecular weights (approx. 50–55 kDa), indicating that they exist predominantly as monomers (fractions X–Z; figure 2*a*). BMP stimulation, which causes an increase in phosphorylation of SMAD1 over basal levels, caused a portion of SMAD1 and phospho-SMAD1 to elute in slightly higher-molecular-weight fractions (fractions V–W as well as X–Z; figure 2*b*). TGF-β stimulation, which induces phosphorylation of SMAD2 and SMAD3, caused a more dramatic change in the elution profile of phospho-SMADs 2 and 3, which were now detected in fractions corresponding to much higher molecular weights (fractions T–Y; figure 2*c*).

Consistent with the idea that activated R-SMADs form a complex with SMAD4 [15], BMP-induced phospho-SMAD1 (figure 2*b*) and, in particular, TGF-β-induced phospho-SMADs 2 and 3 (figure 2*c*) eluted in fractions that overlapped with those containing SMAD4 (fractions T–X; figure 2*b*,*c*). Surprisingly, the elution profile of SMAD4 itself was unchanged by BMP or TGF-β stimulation, suggesting that SMAD4 exists in an oligomeric state with itself or with other proteins prior to formation of active R-SMAD/SMAD4 complexes (figure 2*a–c*).

In extracts from untreated, BMP-treated and TGF-β-treated cells, PAWS1 (whose predicted molecular weight is 91 kDa) eluted in fractions corresponding to greater than 670 kDa (predominantly fractions O and P; figure 2*a–c*). A portion of phospho-SMAD1 was also detected in these fractions, irrespective of whether cells had been untreated or treated with BMP or TGF-β. The presence of total SMAD1 was confirmed by immunoblotting SMAD1 IPs from these fractions (the electronic supplementary material, figure S1*b*). PAWS1 elution did not overlap with that of SMAD4- or of TGF-β-induced phospho-SMADs 2 or 3 (figure 2*a,c*). This is consistent with our observations that PAWS1 does not interact with SMAD2 or 3 (figure 1*b*,*e*) or SMAD4 (the electronic supplementary material, figure S1*b*). Together, these findings suggest that a portion of SMAD1 forms a macromolecular complex with PAWS1 that is independent of SMAD4.

### 3.3. PAWS1 does not affect the extent or kinetics of bone morphogenetic protein-induced SMAD1 phosphorylation

**Figure 3.**
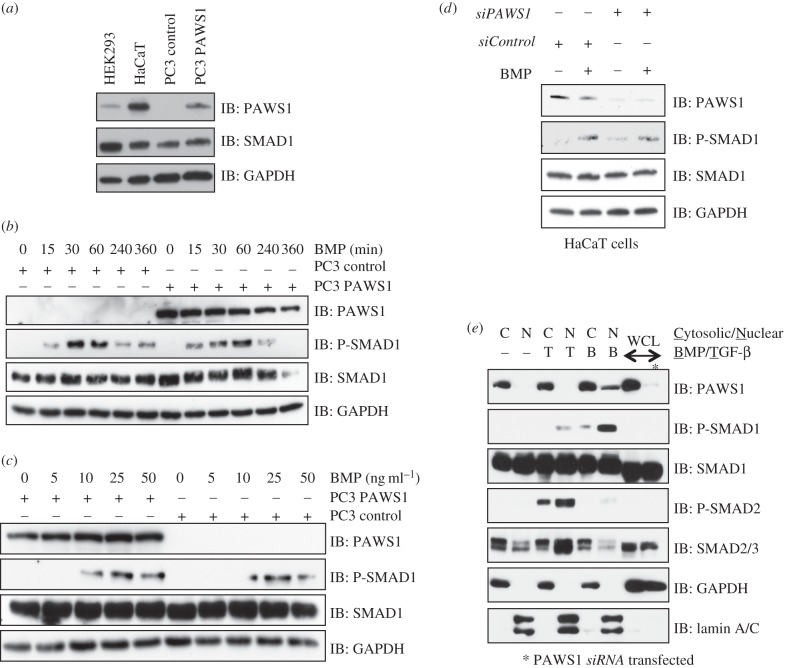
Effect of PAWS1 on BMP-induced SMAD1 phosphorylation. (*a*) Extracts (20 μg protein) from either HEK293, HaCaT, PC3-control (PC3 cells stably integrated with a control vector), or PC3-PAWS1 cells (PC3 cells stably integrated with a vector encoding wild-type PAWS1) were resolved by SDS–PAGE and analysed by immunoblotting using the indicated antibodies. (*b*) PC3-control and PC3-PAWS1 cells were treated with BMP-2 (25 ng ml^−1^) for the indicated time (min) prior to lysis. Extracts (20 μg protein) were resolved by SDS–PAGE and analysed by immunoblotting using the indicated antibodies. (*c*) PC3-control and PC3-PAWS1 cells were treated with the indicated concentrations of BMP-2 for 1 h prior to lysis. Extracts (20 μg protein) were resolved by SDS–PAGE and analysed by immunoblotting using the indicated antibodies. (*d*) PAWS1-depleted HaCaT cells (*siPAWS1*) and HaCaT cells expressing FOXO4 *siRNA* (*siControl*) were treated either with or without BMP-2 (25 ng ml^−1^) for 1 h prior to lysis. Extracts (20 µg protein) were resolved by SDS–PAGE and immunoblotted with the indicated antibodies. (*e*) Extracts from HaCaT cells treated with either BMP-2 (25 ng ml^−1^) or TGF-β (50 pM) for 1 h, or left untreated were separated into cytosolic and nuclear fractions. Fractions and whole cell lysates (WCLs) were resolved by SDS–PAGE and analysed by immunoblotting using the indicated antibodies. GAPDH and lamin A/C were used as cytosolic and nuclear controls, respectively.

PAWS1 is expressed in many mouse tissues and in many human cell lines, although not in PC3 prostate cancer cells (the electronic supplementary material, figure S2*a*,*b*). To investigate the role of PAWS1 in BMP signalling, we therefore made use of PC3 and HaCaT cells. Because PC3 cells lack endogenous PAWS1, we stably reintroduced, by retroviral infection, either a control vector (PC3-control) or a vector encoding human PAWS1 (PC3-PAWS1). PC3-control cells did not express PAWS1, and PC3-PAWS1 cells expressed PAWS1 at levels comparable to those seen in HaCaT cells (figure 3*a*).

To explore the effect of PAWS1 on BMP-induced phosphorylation of SMAD1, PC3-control and PC3-PAWS1 cells were treated with BMP and assayed at intervals thereafter (figure 3*b*). In both cell types, BMP induced SMAD1 phosphorylation within 15 min, the levels reaching a maximum by 1 h and falling thereafter (figure 3*b*). PAWS1 had no detectable effect on the kinetics or extent of BMP-induced SMAD1 phosphorylation, or on the levels of SMAD1 protein (figure 3*b*). To ask whether PAWS1 affects cellular sensitivity to BMP signals, PC3-control and PC3-PAWS1 cells were treated with increasing amounts of BMP, and SMAD1 phosphorylation was monitored by immunoblotting. There was no significant difference in the levels of phospho-SMAD1 in the two cell types (figure 3*c*).

To confirm that PAWS1 does not affect BMP-induced phosphorylation of SMAD1, a loss-of-function study was performed. HaCaT cells were transfected with siRNA oligonucleotides targeting PAWS1 or (as a control) FOXO4, and treated with BMP. In cells transfected with PAWS1 *siRNA*, PAWS1 protein expression was depleted by approximately 90% compared with control. PAWS1 depletion did not significantly alter the levels of phospho-SMAD1 induced by BMP (figure 3*d*).

Treatment of cells with BMP causes the nuclear translocation of phospho-SMAD1 [15]. To examine the subcellular localization of PAWS1, control- or ligand-stimulated HaCaT cells were separated into nuclear and cytosolic fractions. Under basal- or TGF-β-stimulated conditions, PAWS1 was detected predominantly in the cytosolic fractions. However, upon BMP stimulation, a small portion of PAWS1 was detected in the nuclear fraction. As expected, phospho-SMAD1 and phospho-SMAD2 were detected in the nuclear fractions upon BMP and TGF-β stimulations respectively (figure 3*e*). Lamin A/C and GAPDH used as controls were detected in the nuclear fraction and cytosolic fraction respectively (figure 3*e*).

Our attempts to explore the intracellular localization of PAWS1 by immunofluorescence were unsuccessful: neither of our antibodies proved suitable for endogenous immunostaining.

### 3.4. PAWS1 is phosphorylated by type I bone morphogenetic protein receptor *in vitro* and *in vivo*

The observations that SMAD1 and PAWS1 interact in a complex, and that a portion of PAWS1 translocates to the nucleus upon BMP treatment, prompted us to ask whether BMP signalling causes a post-translational modification to occur within PAWS1. We therefore generated HEK293 cells carrying a single copy of GFP-PAWS1 and used mass spectrometry to analyse phospho-modification of material immunoprecipitated from control cells or cells treated with BMP. BMP-treated cells, but not controls, proved to contain a triphosphopeptide corresponding to residues 608–623 (RPSVASSVSEEYFEVR) of human PAWS1. Our mass spectrometric analysis established Ser610 as one of the phosphosites, but was unable to establish the two remaining phosphoresidues within the peptide.

**Figure 4.**
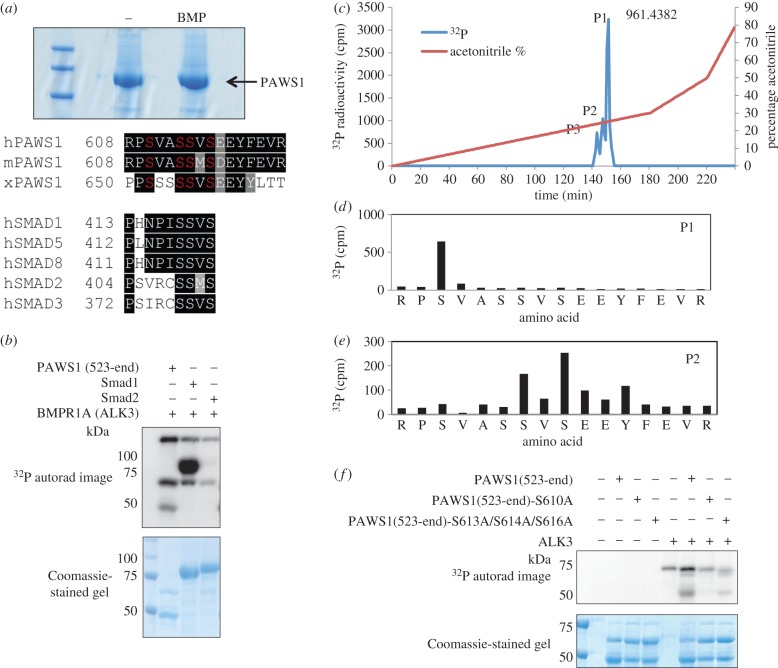
Phosphorylation of PAWS1 by BMPR1A (ALK3). (*a*) GFP IPs from HEK293 cells stably expressing GFP-PAWS1 treated either with or without BMP-2 (25 ng ml^−1^) were resolved by SDS–PAGE. The gel was Coomassie-stained, and bands representing GFP-PAWS1 were excised, digested with trypsin and phosphopeptides identified by mass spectrometry. The sequence alignment of the PAWS1 triphosphopeptide identified upon BMP treatment compared with other vertebrates is shown. Also shown for comparison is the sequence alignment of the SSVS motif in different R-SMADs. h, human; m, mouse; x, *Xenopus laevis.* (*b*) Kinase assays were set up with BMPR1A (ALK3) using GST-SMAD1, GST-SMAD2 and GST-PAWS1 (523-end) as substrates using ^γ32^P-ATP as described in the methods. Samples were resolved by SDS–PAGE, the gel was Coomassie-stained and radioactivity was analysed by autoradiography. (*c*) GST-PAWS1(523-end) phosphorylated by BMPR1A in B was excised, digested with trypsin and resolved by HPLC on a C_18_ column using an increasing acetonitrile gradient as indicated. Three peaks (P1–3) of ^32^P radioactivity release were observed. Analysis of peak P1 by LC–MS–MS revealed the phosphopeptide RPSVASSVSEEYFEVR, with an observed *m*/*z* of 961.4382[2+]. Similarly, peak P2 revealed the diphosphopeptide RPSVASSVSEEYFEVR, with observed *m*/*z* of 1001.42 [2+]. (*d*) Solid-phase sequencing of peak P1 showed the ^32^P radioactivity after the third cycle of Edman degradation. (*e*) Solid-phase sequencing of peak P2 revealed the release of ^32^P radioactivity after the seventh and ninth cycles of Edman degradation. Amino acid sequences in (*d*,*e*) were deduced from LC–MS–MS analysis. (*f*) As in (*b*) except that BMPR1A (ALK3) was incubated in a kinase assay with GST-PAWS1(523-end), GST-PAWS1(523-end)S610A or GST-PAWS1(523-end)S613A/S614A/S616A used as substrates.

We note that this PAWS1 peptide is highly conserved among vertebrate PAWS1 orthologues (figure 4*a*), and that the SSVS motif, corresponding to residues 613–616 of PAWS1, is identical to the SSXS motif at the C-termini of R-SMADs (figure 4*a*). The second and third serine residues within the SSXS motif of SMADs 1, 5 and 8 are phosphorylated by type I BMP receptor kinases, causing their translocation to the nucleus [15]. We therefore reasoned that PAWS1 might be a novel target for BMP type I receptor kinases. To date, no non-SMAD substrates for BMP type I receptor kinases (ALKs 2, 3 and 6) have been reported. To test this idea, we established an *in vitro* kinase assay using a GST-PAWS1(523–823) fragment as a substrate for BMPR1A (ALK3). PAWS1, like SMAD1, was phosphorylated *in vitro* by ALK3, whereas SMAD2, used as a negative control, was not (figure 4*b*). Activated versions of the type I BMP receptors ALK2 and ALK6 also phosphorylated PAWS1 *in vitro* (the electronic supplementary material, figure S3), and this phosphorylation was inhibited by LDN193189, a potent inhibitor of type I BMP receptor kinases [8,31] (the electronic supplementary material, figure S3).

We sought to map the *in vitro* ALK3 phosphorylation sites within PAWS1 by a combination of mass spectrometry and solid-phase Edman sequencing. ^32^P-labelled GST-PAWS1 phosphorylated by ALK3 was digested with trypsin, and the resulting peptides were separated by reverse-phase chromatography on a C_18_ column. Three ^32^P-labelled peaks, one major (P1) and two minor (P2 and P3), eluted at 26%, 25% and 24% acetonitrile, respectively (figure 4*c*). The molecular mass of P1 determined by mass spectrometry (961.4382 Da) corresponded to that of a tryptic phosphopeptide comprising residues 608–623 with a single phosphorylation modification. ^32^P radioactivity was released after the third cycle of Edman degradation (figure 4*d*), confirming that phosphorylation of PAWS1 occurs at Ser610. For P2, ^32^P radioactivity was released after the seventh and the ninth cycles of Edman degradation, consistent with phosphorylation at Ser614 and Ser616 of PAWS1 (figure 4*e*). There was not enough material for analysis of the phosphorylation sites within peak P3.

These results indicate that ALK3 phosphorylates PAWS1 predominantly at Ser610 but can also phosphorylate at Ser614 and Ser616 *in vitro.* Consistent with this conclusion, mutation of Ser610 to Ala almost completely abolished phosphorylation of PAWS1 by ALK3 *in vitro* (figure 4*f*), and the major radioactive peak corresponding to Ser610 (P1 in figure 4*c*) was lost when the tryptic fragments of PAWS1(S610A) phosphorylated by ALK3 were subjected to reverse-phase HPLC as above (the electronic supplementary material, figure S4). Peak P2, corresponding to Ser614/Ser616 phosphorylation on PAWS1, was unaffected (the electronic supplementary material, figure S4), and indeed, this dual Ser614/Ser616 phosphorylation was confirmed by Edman degradation and mass spectrometry (the electronic supplementary material, figure S4). Mutation of Ser613, Ser614 and Ser616 to Ala resulted in a significant but not complete inhibition of phosphorylation of PAWS1 by ALK3 *in vitro* (figure 4*f*). It is Ser614 and Ser616 that correspond to the sites in the SMAD1 SSVS motif that are phosphorylated by ALK3, so it was surprising that PAWS1 is phosphorylated predominantly at Ser610; this is discussed below.

### 3.5. Phosphorylation of PAWS1 at Ser610 regulates the expression of bone morphogenetic protein-dependent SMAD4-independent target genes

**Figure 5.**
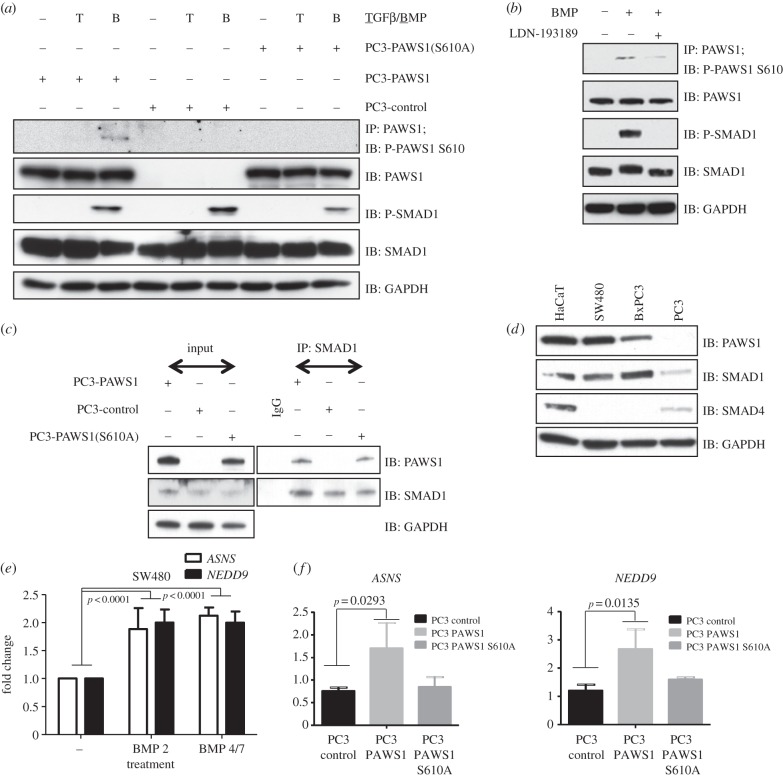
The role of PAWS1 in the BMP pathway. (*a*) PC3-control, PC3-PAWS1 and PC3-PAWS1(S610A) cells were treated with either BMP-2 (25 ng ml^−1^), TGF-β (50 pmol) or left untreated for 1 h prior to lysis. PAWS1 was immunoprecipitated from cell extracts (1 mg protein) using anti-PAWS1 antibody. Anti-PAWS1 IPs and extract inputs (20 µg protein) were resolved by SDS–PAGE and immunoblotted with the indicated antibodies. (*b*) HaCaT cells were either left unstimulated or stimulated with BMP-2 (25 ng ml^−1^) or BMP-2 and LDN193189 (100 nM) for 1 h prior to lysis. PAWS1 was immunoprecipitated from cell extracts (1 mg protein) using anti-PAWS1 antibody. Anti-PAWS1 IPs and extract inputs (20 µg protein) were resolved by SDS–PAGE and immunoblotted with the indicated antibodies. (*c*) PC3-control, PC3-PAWS1 and PC3-PAWS1(S610A) cells were lysed and SMAD1 immunoprecipitated from extracts (1 mg protein) using anti-SMAD1 antibody. IP using pre-immune IgG was used as control from PC3-PAWS1 cell extracts (1 mg protein). SMAD1 IPs, IgG IP and extract inputs (20 µg protein) were resolved by SDS–PAGE and immunoblotted with the indicated antibodies. (*d*) Extract inputs (20 μg protein) from HaCaT, SW480, BxPC3 and PC3 cells were resolved by SDS–PAGE and analysed by immunoblotting using the indicated antibodies. (*e*) SW480 cells were either treated with BMP-2 (25 ng ml^−1^) and BMP-2/7 (10 ng ml^−1^ each) or left untreated for 6 h prior to RNA isolation. The relative expression of the indicated genes was analysed by qRT-PCR as described in the methods. The results show the fold change in gene expression relative to unstimulated controls. Data are represented as mean of three biological replicates and error bars indicate standard deviation (*n* = 3). (*f*) PC3-control, PC3-PAWS1 and PC3-PAWS1(S610A) cells were either treated with BMP-2 (25 ng ml^−1^) or left untreated for 6 h prior to RNA isolation. The relative expression of the indicated genes was analysed by qRT-PCR as described in §5. The results show the fold change in gene expression relative to unstimulated controls. Data are represented as mean of three biological replicates and error bars indicate standard deviation (*n* = 3).

To investigate the significance of BMP-induced phosphorylation of PAWS1 *in vivo*, we raised a phospho-specific antibody recognizing PAWS1 phosphorylated at Ser610 (PAWS1-S610P; figure 5*a*). We also generated PC3 cells stably integrated with a PAWS1(S610A) mutant. In PC3-PAWS1 cells, treatment with BMP, but not TGF-β, resulted in the phosphorylation of PAWS1 at Ser610, as detected by our phospho-specific antibody. By contrast, the PAWS1-S610P antibody did not detect a product in PC3-control cells or in PC3-PAWS1(S610A) cells, confirming the specificity of this reagent (figure 5*a*). The introduction of wild-type or the S610A mutant version of PAWS1 in PC3 cells did not significantly alter the levels of BMP-induced phospho-SMAD1 (figure 5*a*).

We next asked whether BMP induces the phosphorylation of endogenous PAWS1 at Ser610 in HaCaT cells. Treatment of HaCaT cells with BMP indeed caused phosphorylation of PAWS1 at Ser610, and this was inhibited by LDN-193189 (figure 5*b*). The time-course of BMP-induced PAWS1 phosphorylation mirrored that of the phosphorylation of the tail of SMAD1 (the electronic supplementary material, figure S5). Interestingly, phosphorylation of Ser610 of PAWS1 does not affect its ability to interact with SMAD1 (figure 5*c*).

BMP signalling regulates target gene expression in SMAD4-dependent and -independent manners [23,32]. For example, BMP induces *ID1* and *SnoN* in an SMAD4-dependent manner [32], whereas genes such as *NEDD9* and *ASNS* can be activated in cells lacking SMAD4 (figure 5*d,e* and the electronic supplementary material, figure S6*b*; [23]). Because PAWS1 forms a complex with SMAD1 independent from SMAD4, we reasoned that induction of SMAD4-independent BMP target genes might occur through PAWS1. Consistent with this suggestion, BMP induced *NEDD9* and *ASNS* expression in PC3-PAWS1 cells, but not in PC3-control cells and not in PC3-PAWS1(S610A) cells, further suggesting that phosphorylation of PAWS1 at Ser610 is necessary for BMP-induced activation of these genes (figure 5*f*). Expression of BMPR2 was unaffected by the presence of wild-type PAWS1 or the S610A mutant in PC3 cells (the electronic supplementary material, figure S6*a*). BMP-induced expression of the SMAD4-dependent target gene *ID1* was not affected significantly by restoration of wild-type PAWS1 expression in PC3 cells (the electronic supplementary material, figure S7*c*).

### 3.6. PAWS1 regulates the expression of non-bone morphogenetic protein target genes

**Figure 6.**
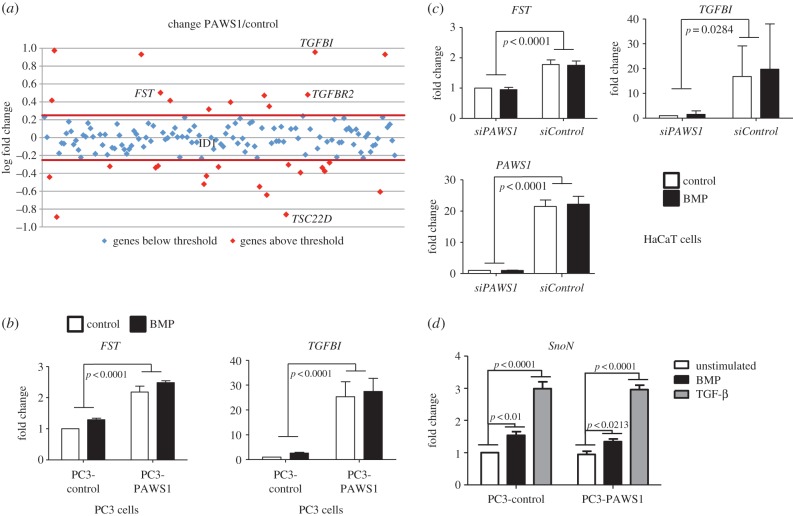
PAWS1 impacts the expression of multiple genes in the TGF/BMP pathways independent of BMP treatment. (*a*) Scatter plots of log fold change in expression in PC3-PAWS1 over PC3-control cells of 150 TGF-β/BMP pathway components and target genes analysed by qPCR macroarray. Each dot represents the expression of a single gene. (*b*) PC3-PAWS1 and PC3-control cells were treated either with or without BMP-2 (25 ng ml^−1^) for 6 h prior to lysis and the expression of *FST* and *TGFBI* was analysed by qRT-PCR as described in the methods. The results show the fold change in gene expression relative to the levels observed for unstimulated PC3-control cells. Data are represented as mean of three biological repeats and error bars indicate standard deviation (*n* = 3). (*c*) PAWS1-depleted HaCaT cells (*siPAWS1*) or HaCaT cells expressing FOXO4 *siRNA* (*siControl*) were treated with or without BMP-2 (25 ng ml^−1^) for 6 h prior to lysis and the expression of *FST*, *TGFBI* and *PAWS1* was analysed by qRT-PCR. The results show the fold change in gene expression relative to the levels observed for unstimulated *siPAWS1* HaCaT cells. Data are represented as mean of three biological repeats and error bars indicate standard deviation (*n* = 3). (*d*). PC3-control and PC3-PAWS1 cells were treated with control, BMP-2 (25 ng ml^−1^) or TGFβ (50 pM) for 6 h prior to lysis, and the expression of *SnoN* was analysed by qRT-PCR. The results show the fold change in *SnoN* expression relative to the levels observed for control-stimulated PC3-control cells. Data are represented as mean of three biological repeats and error bars indicate standard deviation (*n* = 3).

To explore further the ability of PAWS1 to regulate gene expression, we asked whether the introduction of PAWS1 into PC3 cells regulates the expression of 155 known TGF-β/BMP target genes (figure 6*a* and the electronic supplementary material, figure S7*a,b*). Expression of 20 genes proved to be changed by more than twofold upon restoration of PAWS1 (figure S7*a* and the electronic supplementary material, S5*a,b*). Among these, we confirmed by RT-PCR that expression of *FST*, *TGFBI* and *TGFBR2* was augmented, whereas expression of *TSC22D* was diminished (figure 6*b* and the electronic supplementary material, figure S7*c*).

To ensure that these changes in gene expression were directly linked to PAWS1, we depleted PAWS1 in HaCaT cells by *RNAi* and confirmed that expression of both *FST* and *TGFBI* were reduced (figure 6*c*). However, we also found that BMP treatment of PC3-control or PC3-PAWS1 cells did not alter expression of *FST*, *TGFBI*, *TGFBR2* or *TSC22D* (figure 6*c* and the electronic supplementary material, S6*c*). Similarly, BMP treatment did not affect the expression of *FST* and *TGFBI* in control HaCaT cells or those expressing PAWS1 *siRNA* (figure 6*c*). These results suggest that PAWS1 also regulates gene expression in a manner that is independent of BMP treatment. This is discussed below. We also tested the effect of PAWS1 on the expression of a canonical TGF-β and BMP target gene, *SnoN*. The expression of *SnoN* induced by BMP or TGF-β was identical in both PC3-control and PC3-PAWS1 cells, implying that PAWS1 had no effect on the expression of *SnoN* (figure 6*d*).

## 4. Discussion

Our experiments show that PAWS1 forms a complex with SMAD1 in a SMAD4-independent manner; that it is a target of type I BMP receptor kinases (and is the first such non-SMAD target to be identified); and that it is a novel player in the BMP signal transduction pathway. Of particular significance, PAWS1 regulates the expression of some SMAD4-independent BMP target genes as well as some BMP-independent genes.

### 4.1. A non-canonical PAWS1–SMAD1 complex

PAWS1 interacts with SMAD1 but not with SMAD2/3. Although the linker domain of the R-SMADs is the least conserved region, it is the SMAD1-MH2 domain that mediates the interaction with PAWS1 and presumably provides the observed specificity. Interaction of the R-SMADs with SMAD4 to form an active complex [15] occurs following the ligand-induced phosphorylation of R-SMADs at the SXS motif within the MH2 domain. Further work is required to understand the nature of the interaction between PAWS1 and the MH2 domain of SMAD1.

Most SMAD1 in our cells forms a ‘canonical’ complex with SMAD4 upon BMP treatment. However, in both control- and BMP-treated extracts, a subfraction of SMAD1 associates with PAWS1 in a high-molecular-weight complex that does not include SMAD4. We do not know the identity of the other proteins in this complex, but our results suggest that it plays a hitherto unrecognized role in BMP signalling. The interaction between PAWS1 and SMAD1 is not affected by treatment of cells with BMP or TGF-β, suggesting that the association is constitutive. BMP treatment of cells causes some PAWS1 to translocate to the nucleus. This nuclear accumulation of PAWS1 may occur through interaction with SMAD1: BMP can induce the nuclear localization of phosphorylated SMAD1 even in the absence of SMAD4 [33].

### 4.2. PAWS1: the first non-SMAD type I bone morphogenetic protein receptor substrate

Immunoprecipitation of PAWS1 from BMP-treated cell extracts allowed the identification of a triphosphopeptide that includes an SSVS motif that is present in SMAD1 and which, in that molecule, is phosphorylated by the type I BMP receptor kinase. No non-SMAD substrates for type I BMP receptor kinases have previously been reported, so it is significant that BMPR1A (ALK3) phosphorylated PAWS1 at Ser610, Ser614 and Ser616 *in vitro*. Comparison with the SSVS motif of SMAD1 would predict that the major phosphorylation site of PAWS1 would be Ser614 and Ser616, so it was surprising that Ser610 was the major PAWS1 phosphorylation site. Nevertheless, we go on to show that PAWS1 is also phosphorylated at Ser610 in response to BMP *in vivo*, and that Ser610 is necessary for the activation of SMAD4-independent BMP target genes such as *NEDD9* and *ASNS* (see below).

The implication that type I BMP and TGF-β receptor kinases (ALKs 1–7) have substrates other than SMADs is consistent with knockout studies in mice, where the loss of ALKs 2, 3 or 6 result in phenotypes that cannot fully be explained simply by the failure to activate SMADs 1, 5 or 8 [34–38]. There are likely to be many more non-SMAD substrates for type I BMP and TGF-β receptor kinases.

### 4.3. PAWS1 and the bone morphogenetic protein signalling pathway

The absence of SMAD4 in the complex that contains PAWS1 and SMAD1 suggests that PAWS1 may play a unique function in the BMP signalling pathway. Consistent with this notion, PAWS1 does not influence BMP-induced phosphorylation of SMAD1 or the expression of SMAD4-dependent BMP target genes such as *ID1* and *SnoN*. However, the activation of *NEDD9* and *ASNS* in response to BMPs was lost in PC3 cells lacking PAWS1 and was restored upon the reintroduction of wild-type PAWS1 but not the PAWS1(S610A) mutant. We note that *NEDD9* has been implicated in cellular migration as well as in the invasion and metastasis of cancer cells [39,40], and that unregulated *ASNS* expression has also been linked with cancer [41]. It will be interesting to discover whether the expression of *PAWS1* itself is misregulated in cancer.

### 4.4. PAWS1: beyond the bone morphogenetic protein signalling pathway

Our analysis of 155 known TGF-β/BMP target genes indicates that several of these are differentially expressed upon reintroduction of PAWS1 in PC3 cells, and that this occurs in a ligand-independent fashion. These observations were confirmed for the genes *FST* and *TGFBI* in PC3 cells following introduction of PAWS1 and in HaCaT cells following depletion of PAWS1 by *RNAi*. PAWS1 therefore regulates gene expression independent from BMP signalling as well as in a ligand-dependent manner, and a global transcriptomic analysis of genes affected by PAWS1 may yield clues to possible biochemical roles beyond the BMP pathway.

To complement such an analysis, it will be necessary to understand more about PAWS1 as a protein. Sequence analysis offers few functional clues beyond the presence of a putative pseudo-phospholipase D (PLD) active site motif, but we note that PLD activity was not detected in the related proteins FAM83A and B [25,26]. It is, nevertheless, possible that PAWS1 interacts with phospholipids and/or other PLDs, or that it acts as a scaffolding protein to control signalling pathways downstream of PLDs. Uncovering the precise functional roles of PAWS1 will enable us to ask how BMP signalling and SMAD1 impact on the biochemical properties of PAWS1.

## 5. Material and methods

### 5.1. General

A PAWS1 antibody was generated by injecting GST-PAWS1 (amino acids 715–815) into sheep. The P-PAWS1 S610 antibody was generated by injecting peptide GPGPRRPS*VAS (* denotes phospho-Ser) into rabbit. The antibodies were subsequently affinity purified. Anti-FLAG-M2-horseradish peroxidase (HRP) antibody was from Sigma. HA-HRP antibody was from Roche. Antibodies recognizing phospho-SMAD1/5/8, phospho-SMAD2, phospho-SMAD3, SMAD2/3, GAPDH and lamin A/C were from Cell Signalling Technology. HRP-conjugated secondary antibodies and light-chain-specific HRP-conjugated antibodies were from Jackson Laboratories. BMP-2 and BMP-4/7 were from R&D Systems. The nuclear cytoplasmic extraction kit was from Thermo Scientific. The first strand cDNA synthesis kit was from Invitrogen. 2X SYBR green master mix was from BioRad. pBABE-Puro, pCMV-Gag-Pol and pCMV-VSVG constructs were from Cell Biolabs. All plasmids for mammalian cell expression were cloned into pCMV5, pBABE-puro or pcDNA-FRT-TO vectors with N-terminal FLAG, HA or GFP tags as indicated. For bacterial expression of proteins, SMAD1, SMAD2 and PAWS1 (523-end; other mutants) were cloned into pGEX6T vectors. All DNA constructs were verified by DNA sequencing (by the DNA Sequencing Service at University of Dundee; www.dnaseq.co.uk). Bacterial protein expression in BL21 cells and purification were performed as described previously [31].

### 5.2. Cell culture, transfection and lysis

Unless stated otherwise, prostate cancer-derived PC3 cells, human embryonic kidney HEK293 cells, HeLa cells, SW480 cells, BxPC3 cells and human keratinocyte HaCaT cells were propagated in DMEM supplemented with 1% penicillin/streptomycin, 2 mM l-glutamine (Gibco) and 10% FBS (Hyclone). Cells were kept at 37°C in a humidified incubator with 5% CO_2_. pcDNA-FRT-TO plasmids encoding GFP- or GFP-tagged PAWS1 were used to generate stable tetracycline-inducible FlpIN-TRex (Invitrogen) HEK293 and U2OS cell lines following the manufacturer's protocol. The cells were grown in medium that additionally contained 100 µg ml^−1^ hygromycin and 15 µg ml^−1^ blasticidin as described previously [42]. For overexpression of pCMV5 plasmids encoding FLAG- or HA-tagged proteins, HEK293 cells were transfected using PEI as described previously [43]. The *siRNA* oligonucleotides (300 pmole total/10-cm diameter dish) were transfected into HaCaT cells using Transfectin (BioRad). Cells were harvested 48 h post-transfection. For protein analysis, cells were scraped directly with cell lysis buffer (50 mM Tris–HCl pH 7.5, 1 mM EGTA, 1 mM EDTA, 1% Triton X-100, 1 mM activated sodium orthovanadate, 50 mM sodium fluoride, 5mM sodium pyrophosphate, 0.27 M sucrose, 5 mM β-glycerophosphate, 0.1% β-mercaptoethanol and one tablet of protease inhibitor cocktail (per 25 ml)) and snap frozen in liquid nitrogen.

### 5.3. Generation of PC3 cells stably expressing wild-type PAWS1 or PAWS1-S610A mutant

Retroviral pBABE-puromycin control vector (1 µg each) or vectors encoding PAWS1 and PAWS1-S610A mutant were co-expressed with CMV-Gag/Pol (0.9 µg) and CMV-VSVG (0.1 µg) constructs in HEK293T cells. Retroviruses were collected 48 h post-transfection from the culture medium by filtration through 0.45 µm filters into sterile Falcon tubes as described previously [44]. PC3 cells, plated at approximately 40% confluence 24 h previously, were infected by transferring filtered retroviruses directly onto the cells together with 8 µg ml^−1^ polybrene. Twenty-four hours post-infection, cells were cultured in the presence of medium containing puromycin (2 µg ml^−1^) for selection of infected cells.

### 5.4. Immunoprecipitation

Snap frozen cell extracts were allowed to thaw on ice and centrifuged at 14 000 rpm for 10 min at 4°C. Protein concentration was determined spectrophotometrically. Extracts (1 mg unless stated otherwise) were then subjected to immunoprecipitation using 10 µl packed beads (GFP-trap, anti-FLAG-M2 or specific antibody covalently bound to protein G sepharose beads or magnetic Dyna-beads (1 µg antibody per 5 µl packed beads)) in a rotating platform for 2 h at 4°C. IPs were then washed twice in lysis buffer with 0.5 M NaCl, and twice in buffer A (50 mM Tris–HCl pH 7.5, 0.1 mM EGTA, 0.1% β-mercaptoethanol) at 4°C. Samples were reduced in SDS–sample buffer (250 mM Tris–HCl pH 6.8, 10% SDS, 50% glycerol, 0.1% bromophenol blue; 0.1% β-mercaptoethanol), boiled for 5 min and resolved by SDS–PAGE.

### 5.5. Mass spectrometry

FLAG control or a FLAG-SMAD1(L + MH2) fragment expressed in HEK293 cells was isolated from cleared extracts (50 mg protein) by immunoprecipitation with anti-FLAG-M2 antibody coupled to agarose beads. The IPs were washed and incubated with cleared HeLa extracts (100 mg) at 4°C for 4 h. After washing, FLAG-proteins and any interacting partners were eluted using 3X FLAG peptide following the manufacturer's protocol. Eluted proteins were reduced in sample buffer and resolved by SDS–PAGE. Coomassie-stained bands were excised and tryptically digested. Mass spectrometric analysis of the resulting peptides was performed by LC–MS–MS as described previously [43].

### 5.6. Gel filtration chromatography

Extracts from HaCaT cells treated with or without the indicated ligands were cleared by centrifugation and further cleared through Spin-X tubes. Protein extract (1 mg) was subjected to separation through a Superose 6 10/300 GL column (GE Healthcare), which was washed and equilibrated with buffer containing 50 mM Tris–HCl 7.5, 150 mM NaCl, 0.03% Brij-35. Thirty-two fractions were collected, and they were processed as described previously [43].

### 5.7. Immunoblotting

Cell extracts were cleared by centrifuging at 14 000 rpm for 10 min at 4°C. Extracts (20 µg) or IPs (30% of total unless stated otherwise) were reduced in SDS sample buffer and boiled for 5 min, resolved using SDS–PAGE and transferred onto PVDF membranes. Membranes were blocked with 5% non-fat dry milk powder in TBST (50 mM Tris, 150 mM NaCl, 0.2% Tween-20) incubated overnight at 4°C with primary antibody, followed by incubation with an HRP-conjugated secondary antibody (1 : 10 000). Antigen–antibody complexes were detected with enhanced chemiluminescence reagents.

### 5.8. Quantitative PCR

Real-time quantitative reverse transcription PCR (qRT-PCR) was carried out using 1 µg of isolated RNA and the SuperScript cDNA kit (Invitrogen) according to the manufacturer's protocol. qRT-PCRs were performed in triplicate (10 µl) according to the manufacturer's protocol, including forward and reverse primers (0.5 µM each), 50% SYBR green master mix (BioRad) and a cDNA equivalent of 1 ng µl^−1^ RNA in a CFX 384 real-time system qRT-PCR machine (BioRad). The data were normalized to the geometrical mean of two housekeeping genes (*GAPDH* and *HPRT1*) and analysed by the Pfaffl method [45].

### 5.9. RNAi and qRT-PCR primers

Primers were designed using PerlPrimer and purchased from Invitrogen. Primers (5′–3′): PAWS1 forward: CACAGAAGGTGATAGCTGTG; reverse: ACTTGACGTTACTCTCATCCA; FOXO4 forward: TTGGAGAACCTGGAGTGTGACA; reverse: AAGCTTCCAGGCATGACTCAG; ID1 forward: AGGCTGGATGCAGTTAAGGG; reverse: GGTCCTTTTCACCAGCAAGCT; GAPDH forward: TGCACCACCAACTGCTTAGC; reverse: GGCATGGACTGTGGTCATGAG; HPRTI forward: TGACACTGGCAAAACAATGCA; reverse: GGTCCTTTTCACCAGCAAGCT; NEDD9 forward: GCTCTATCAAGTGCCAAACCC; reverse: GGTTCCCCCAATGCTTCTCT; ASNS forward: AACTGCTGCTTTGGATTTCAC; reverse: GCTGTTGCATCTTCTTATGGT; BMPR2 forward: TGGAACATACCGTTTCTGCT; reverse: GAATGAGGTGGACTGAGTGG; TGFBI forward: ATCACCAACAACATCCAGCA; reverse: CCGTTACCTTCAAGCATCGT; FST forward: GATCTTGCAACTCCATTTCGG; reverse: GGCTATGTCAACACTGAACAC; TGFBR2 forward: GCTGTATGGAGAAAGAATGACGA; reverse: ACAGGAACACATGAAGAAAGTC; TSC22D1 forward: CTATCAGTGGTGACAGTGGG; reverse: TTCACTAGATCCATAGCTTGCTC; SnoN forward: GAGGCTGAATATGCAGGACAG; reverse: CTATCGGCCTCAGCATGG.

*siRNA* against PAWS1 were purchased from Qiagen and targeted to the following sequences: *siRNA* PAWS1-1: AAGATGATGACGACTACGTAA (catalogue no. SI03683897).

*siRNA* PAWS1-2: CCGGGCTAGCGTCTACATGCA (catalogue no. SI03683904).

*siRNA* against FOXO4 was purchased from Eurofins and targeted to the following sequence: *siRNA* FOXO4: CCCGACCAGAGAUCGCUAA.

### 5.10. Statistical analysis

Data are presented as the mean ± s.d. The statistical significance of differences between experimental groups was assessed using the two-way analysis of variance test with Bonferroni post-tests. Differences in means were considered significant if *p* < 0.05.

### 5.11. Analysis of ^32^P-labelled phosphorylation sites

For kinase assays, 20 µl reactions were set up consisting of 150 ng of kinase (GST-ALK3; GST-ALK2 or GST-ALK6; all from Carna Biosciences) and 2 µg substrate protein (GST-SMAD1, GST-SMAD2, GST-PAWS1 (523-end) or other mutants of PAWS1 as indicated) in a buffer containing 50 mM Tris–HCl pH 7.5, 0.1% 2-mercaptoethanol, 0.1 mM EGTA, 10 mM MgCl_2_, 0.5 µM microcystein-LR and 0.1 mM γ^32^P-ATP (500 cpm pmole^−1^ for routine autorad analysis; 10000 cpm pmole^−1^ for mapping phosphoresidues). Assays were performed at 30°C for 30 min and stopped by adding 1× SDS sample buffer and heating to 95°C for 5 min. The samples were resolved by SDS–PAGE, the gels stained with Coomassie blue and dried. Radioactivity was analysed by autoradiography. Identification of the phosphoresidues within PAWS1 was performed as described [46] except that mass spectrometric analysis of phosphopeptides was performed as above for fingerprinting with the addition of multi-stage activation during the MS2 analysis.

### 5.12. Cellular fractionation

Nuclear/cytosolic fractionation was performed using the nuclear and cytosolic extraction kit from Thermo Scientific according to the manufacturer's instructions. Proteins were denatured by boiling for 5 min in SDS sample buffer prior to SDS–PAGE.

## Supplementary Material

Supplementary Figure Legends

## Supplementary Material

Supplementary Figures
