# Supplementary material for: Protein associated with SMAD1 (PAWS1/FAM83G) is a substrate for type I bone morphogenetic protein receptors and modulates bone morphogenetic protein signalling
Source: Open Biol. 2014 Feb 19;4(2):130210. doi: 10.1098/rsob.130210 (PMC3938053; doi:10.1098/rsob.130210)
Supplement: Supplementary Figure Legends [file rsob130210supp1.docx]

**Protein Associated With SMAD1 (PAWS1/FAM83G) is a substrate for type I BMP receptors and modulates BMP signalling**

Janis Vogt^1^, Kevin S. Dingwell^2^, Lina Herhaus^1^, Robert Gourlay^1^, Thomas Macartney^1^, David Campbell^1^, James C. Smith^2^, Gopal P. Sapkota^1*^

^1^MRC Protein Phosphorylation and Ubiquitylation Unit, University of Dundee, Dow Street, Dundee, DD1 5EH, Scotland, UK. ^2^Division of Systems Biology, MRC National Institute for Medical Research, The Ridgeway, Mill Hill, London NW7 1AA, UK.

^*^Address correspondence to: g.sapkota@dundee.ac.uk

**Supplementary Results:**

**PAWS1 expression in cells and tissues**

PAWS1 has not been previously characterised. Therefore prior to investigating potential roles of PAWS1 in the BMP pathway, we assessed its expression in mouse tissues as well as various cell lines by immunoblotting. High levels of PAWS1 protein expression was observed in extracts derived from liver, adipose and pancreas (Figure S2A). Some expression at predicted molecular weight of PAWS1 was detected in brain, lung, kidney, thymus, testis and ovary tissue extracts (Figure S2A, high exposure). A higher molecular weight cross-reacting band was also detected with anti-PAWS1 antibody in brain and liver tissue extracts (Figure S2A). No PAWS1 expression was detected in heart, skeletal muscle and epididymis extracts (Figure S2A). There was no synergistic expression of PAWS1 and SMAD1 in the tissue extracts (Figure S2A). PAWS1 expression was detected in several human cell lines including breast cancer MDA-MB231, osteosarcoma U2OS, HEK293, colon carcinoma RKO, human keratinocyte HaCaT, HeLa, breast cancer MCF7 and human myeloid leukemia KBM-7 (Figure S2B). Interestingly, prostate cancer cells PC3 lacked the endogenous expression of PAWS1 (Figure S2B).

**Figure S1:** PAWS1-SMAD1 interactions. **(A)** HEK293 cells were transfected with GFP or the indicated GFP-SMAD constructs together with FLAG-PAWS1. The extracts and anti-FLAG IPs were analysed by immunoblotting using the indicated antibodies. **(B)** HEK293 cells stably expressing GFP-SMAD4 under tetracycline induction were transfected with constructs encoding either HA-SMAD1 or FLAG-PAWS1 or both. Expression of GFP-SMAD4 was induced by 20 ng/ml doxycycline for 20 h. Cells were treated either with or without BMP-2 (25 ng/ml) for 1 h prior to lysis. Extracts and anti-GFP IPs were analysed by immunoblotting using the indicated antibodies. **(C)** HEK293 cells were transfected with constructs encoding either FLAG, FLAG-SMAD1 or indicated FLAG-SMAD1 truncation mutants together with HA-PAWS1. The extracts and anti-FLAG IPs were analysed by immunoblotting using the indicated antibodies. **(D)** SMAD1 IPs from gel filtration chromatography fractions 16 from Figure 2 were analysed by immunoblotting using SMAD1 antibody.

**Figure S2:** Expression of PAWS1 in mouse tissues and human cell lines. (A) Extracts (20 μg protein) from the indicated mouse tissues were resolved by SDS-PAGE and analysed by immunoblotting using the indicated antibodies. Extended exposure (PAWS1 high) revealed expression of PAWS1 in brain, lung, kidney, thymus, testis and ovary tissue extracts. (B) Extracts (20 μg protein) from the indicated human cell lines were analysed by immunoblotting using the indicated antibodies.

**Figure S3:** LDN-193189 inhibits the phosphorylation of PAWS1 by ALK2 and ALK6 *in vitro.* Kinase assays were setup with either ALK2 or ALK6 and GST-PAWS1(523-end) in the presence of the indicated concentration of LDN-193189. Samples were resolved by SDS-PAGE. The gels were Coomassie-stained, dried and analysed by ^32^P autoradiography.

**Figure S4:** Phosphorylation of PAWS1 S610A by BMPR1A (ALK3). (A) GST-PAWS1 S610A(523-end) phosphorylated by BMPR1A was resolved by SDS-PAGE, excised and then digested with trypsin. The resulting peptides were resolved by HPLC on a C_18_ column using an increasing acetonitrile gradient as indicated. Two peaks (P1-2) of ^32^P radioactivity release were observed. Analysis of peak P1 by LC-MS-MS revealed the diphospho-peptide RPAVASSVSEEYFEVR, observed m/z of 662.6184[3+] while analysis of peak P2 by LC-MS-MS revealed the phospho-peptide RPAVASSVSEEYFEVR, observed m/z of 953.4414[2+] (B) Solid-phase sequencing of peak P1 revealed the release of ^32^P radioactivity after the seventh and ninth cycle of Edman degradation. (C) Solid-phase sequencing of peak P2 revealed the release of ^32^P radioactivity after the ninth cycles of Edman degradation. Amino acid sequences in *B* and *C* were deduced from LC-MS-MS analysis.

**Figure S5:** Kinetics of PAWS1 phosphorylation at Ser610 in response to BMP. HaCaT cells were treated with BMP-2 (25 ng/ml) for the indicated times prior to lysis. PAWS1 was immunoprecipitated from extracts (1 mg protein) using anti-PAWS1 antibody. PAWS1 IPs and extracts (20 μg protein) were resolved by SDS-PAGE and subjected to immunoblotting analysis with the indicated antibodies.

**Figure S6:** The role of PAWS1 in the BMP pathway. (A) BxPC3 cells were either treated with BMP-2 (25 ng/ml), BMP-2/7 (10 ng/ml) or left untreated for 6 h prior to RNA isolation. The relative expression of the indicated genes was analysed by qRT-PCR as described in the methods. The results show the fold change in gene expression relative to unstimulated controls. Data are represented as mean of three biological replicates and error bars indicate standard deviation (n=3). (B) PC3-contol, PC3-PAWS1 and PC3-PAWS1(S610A) cells were either treated with BMP-2 (25 ng/ml) or left untreated for 6 h prior to RNA isolation. The relative expression of the indicated gene was analysed by qRT-PCR as described in the methods. The results show the fold change in gene expression relative to unstimulated controls. Data are represented as mean of three biological replicates and error bars indicate standard deviation (n=3).

**Figure S7:** Impact of PAWS1 on TGF-β/BMP genes. (A) Scatter plots of log fold change of 150 TGF-β/BMP pathway components and target genes analysed by qRT-PCR macroarray using RNA from PC3-control vs. PC3-PAWS1 cell lines. Cells were treated with BMP-2 (25 ng/ml). Each dot represents the expression of a single gene. (B) Table summarising the genes from A that changed in expression by either PAWS1 expression or BMP-2 or both. All genes for which the expression changed by more than 2-fold are listed. Red indicates high and medium expression genes (e.g. qRT-PCR with a Cp value below cycle 30) whereas black indicates low expression genes (e.g. qRT-PCR with a Cp value above cycle 30). (C) PC3-PAWS1 and PC3-control cells were treated either with or without BMP-2 (25 ng/ml) for 6 h prior to lysis. The expression of TGFBR2, TSC22D and ID1 was analysed by qRT-PCR. The results show the fold change in gene expression relative to the levels observed for unstimulated PC3-control cells. Data are represented as mean of three biological repeats and error bars indicate standard deviation (n=3).
