# Supplementary material for: Protein associated with SMAD1 (PAWS1/FAM83G) is a substrate for type I bone morphogenetic protein receptors and modulates bone morphogenetic protein signalling
Source: Open Biol. 2014 Feb 19;4(2):130210. doi: 10.1098/rsob.130210 (PMC3938053; doi:10.1098/rsob.130210)
Supplement: Supplementary Figures [file rsob130210supp2.pptx]

## Slide 1
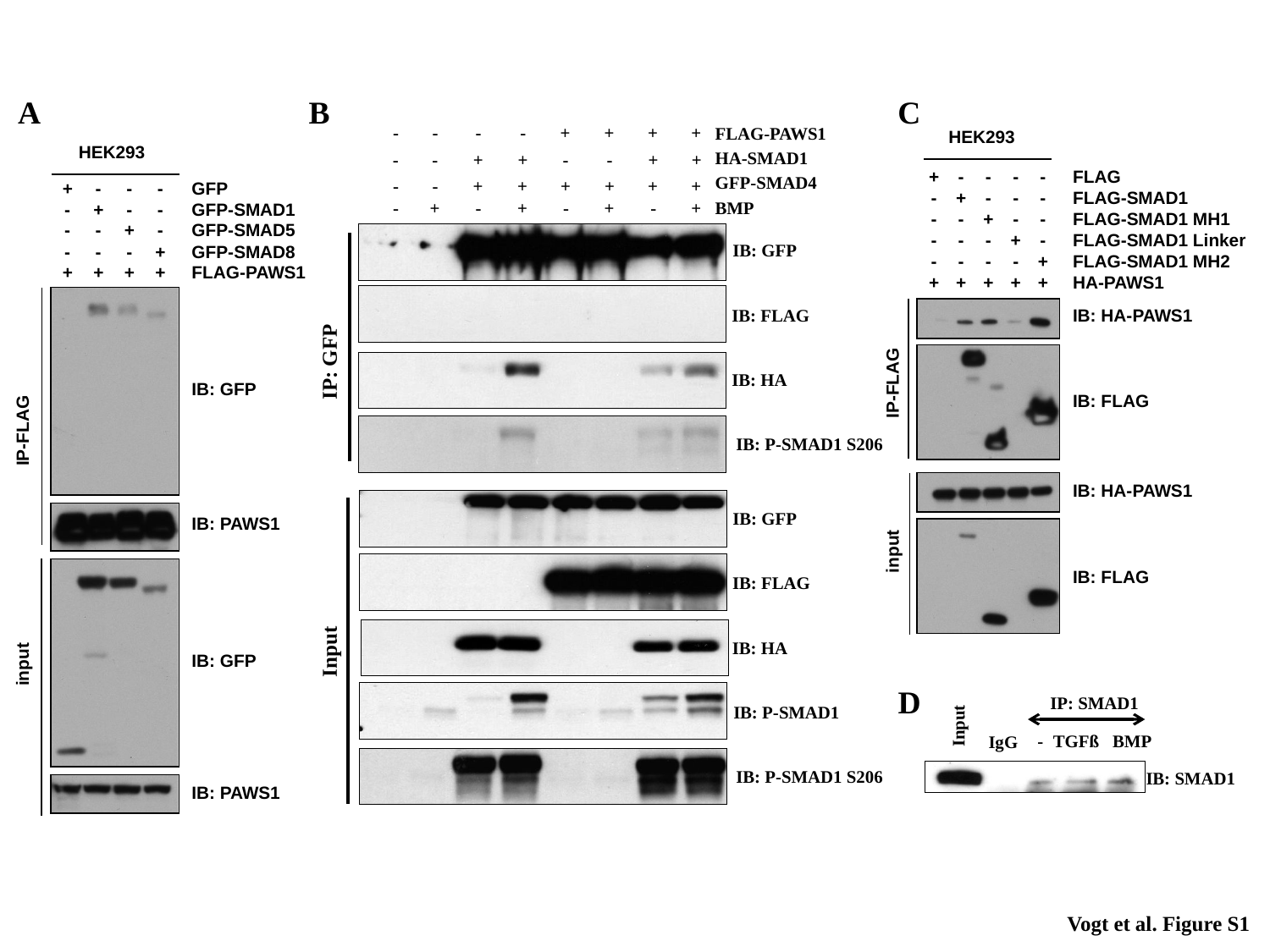

A
B
C
-
-
-
-
+
+
+
+
FLAG-PAWS1
HEK293
HEK293
HA-SMAD1
-
-
+
+
-
-
+
+
+
-
-
-
-
+
-
+
-
-
-
+
-
-
+
-
-
+
-
-
-
+
-
+
-
-
-
-
+
+
FLAG
FLAG-SMAD1
FLAG-SMAD1 MH1
FLAG-SMAD1 Linker
FLAG-SMAD1 MH2
HA-PAWS1
GFP-SMAD4
-
-
+
+
+
+
+
+
+
-
-
-
+
-
+
-
-
+
-
-
+
-
+
-
-
-
+
+
GFP
GFP-SMAD1
GFP-SMAD5
GFP-SMAD8
FLAG-PAWS1
BMP
-
+
-
+
-
+
-
+
IB: GFP
IB: FLAG
IB: HA-PAWS1
IP: GFP
IP-FLAG
IB: HA
IB: GFP
IB: FLAG
IP-FLAG
IB: P-SMAD1 S206
IB: HA-PAWS1
input
IB: GFP
IB: PAWS1
IB: FLAG
IB: FLAG
input
IB: HA
Input
IB: GFP
D
IP: SMAD1
Input
-
TGFß
BMP
IgG
IB: SMAD1
IB: P-SMAD1
IB: P-SMAD1 S206
IB: PAWS1
Vogt et al. Figure S1

## Slide 2
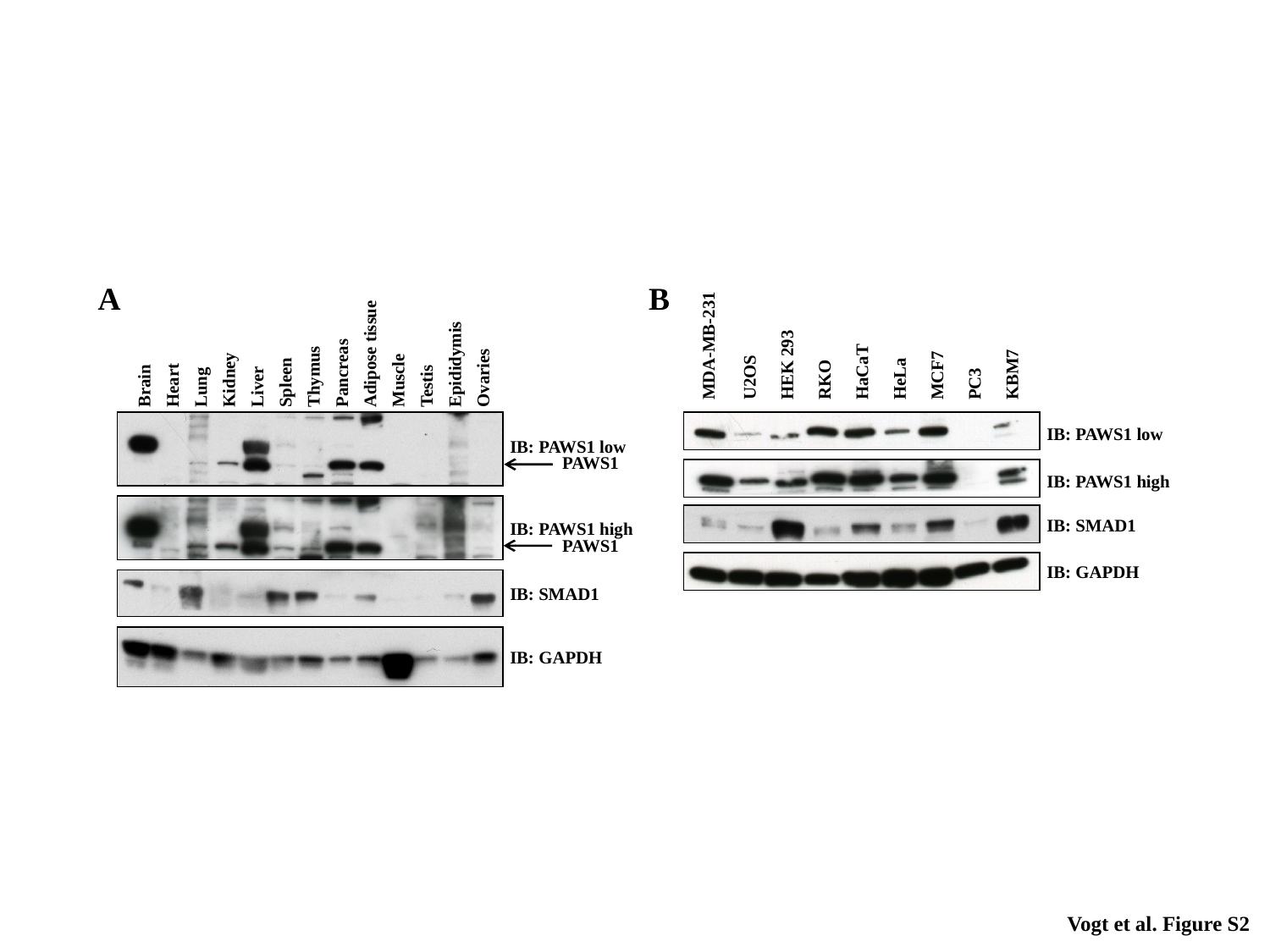

MDA-MB-231
U2OS
HEK 293
RKO
HaCaT
HeLa
MCF7
PC3
KBM7
IB: PAWS1 low
IB: PAWS1 high
IB: SMAD1
IB: GAPDH
Adipose tissue
Pancreas
Epididymis
Muscle
Ovaries
Spleen
Thymus
Kidney
Testis
Heart
Brain
Liver
Lung
IB: PAWS1 low
PAWS1
IB: PAWS1 high
PAWS1
IB: SMAD1
IB: GAPDH
A
B
Vogt et al. Figure S2

## Slide 3
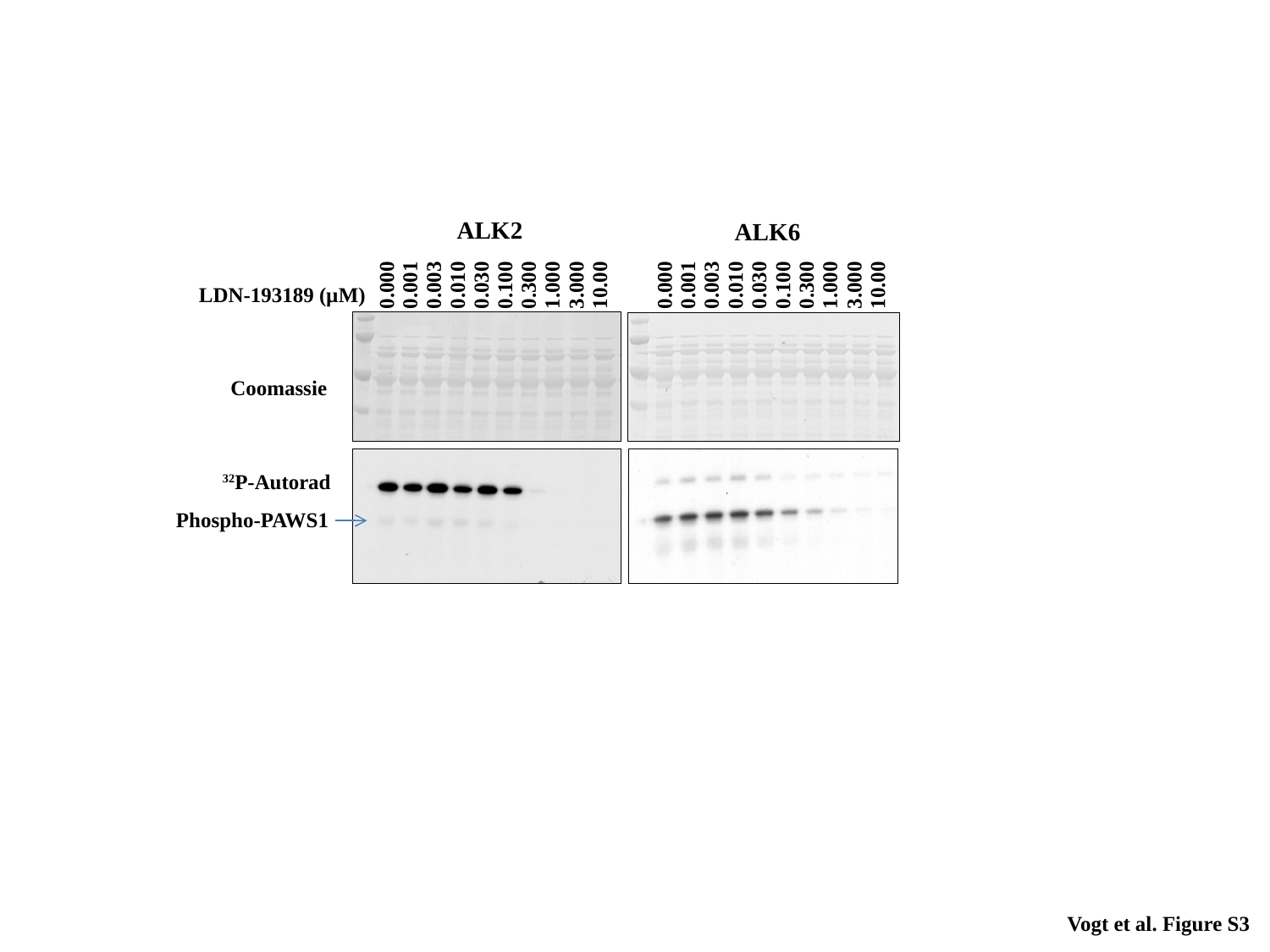

ALK2
ALK6
0.010
0.100
0.100
0.001
0.001
0.010
0.003
0.030
0.300
1.000
3.000
10.00
0.003
0.030
0.300
1.000
3.000
10.00
0.000
0.000
LDN-193189 (µM)
Coomassie
32P-Autorad
Phospho-PAWS1
Vogt et al. Figure S3

## Slide 4
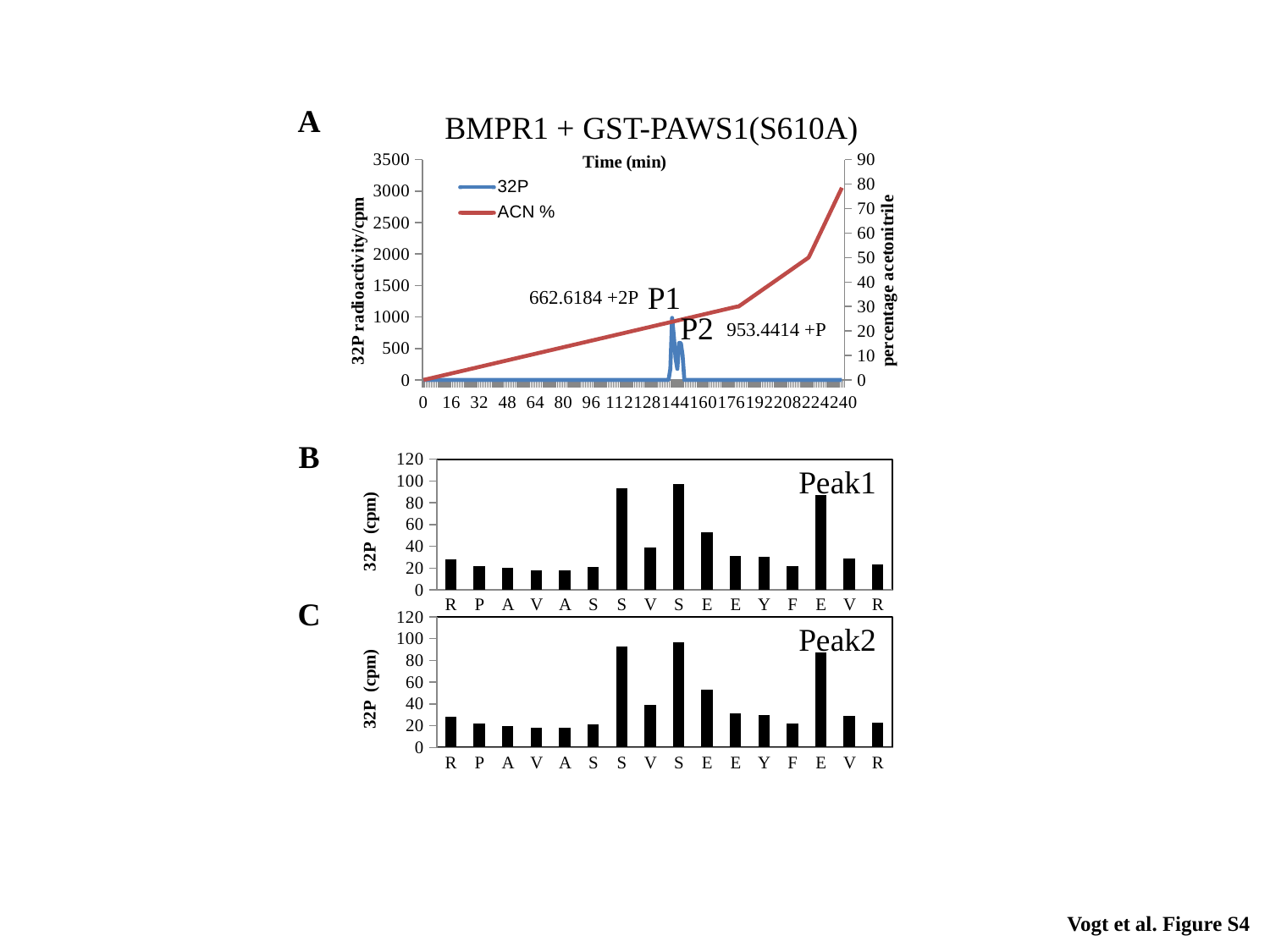

A
BMPR1 + GST-PAWS1(S610A)
[unsupported chart]
P1
662.6184 +2P
P2
953.4414 +P
B
### Chart
| Category | |
|---|---|
| R | 28.0 |
| P | 22.0 |
| A | 20.0 |
| V | 18.0 |
| A | 18.0 |
| S | 21.0 |
| S | 93.0 |
| V | 39.0 |
| S | 97.0 |
| E | 53.0 |
| E | 31.0 |
| Y | 30.0 |
| F | 22.0 |
| E | 87.0 |
| V | 29.0 |
| R | 23.0 |Peak1
C
### Chart
| Category | |
|---|---|
| R | 28.0 |
| P | 22.0 |
| A | 20.0 |
| V | 18.0 |
| A | 18.0 |
| S | 21.0 |
| S | 93.0 |
| V | 39.0 |
| S | 97.0 |
| E | 53.0 |
| E | 31.0 |
| Y | 30.0 |
| F | 22.0 |
| E | 87.0 |
| V | 29.0 |
| R | 23.0 |Peak2
Vogt et al. Figure S4

## Slide 5
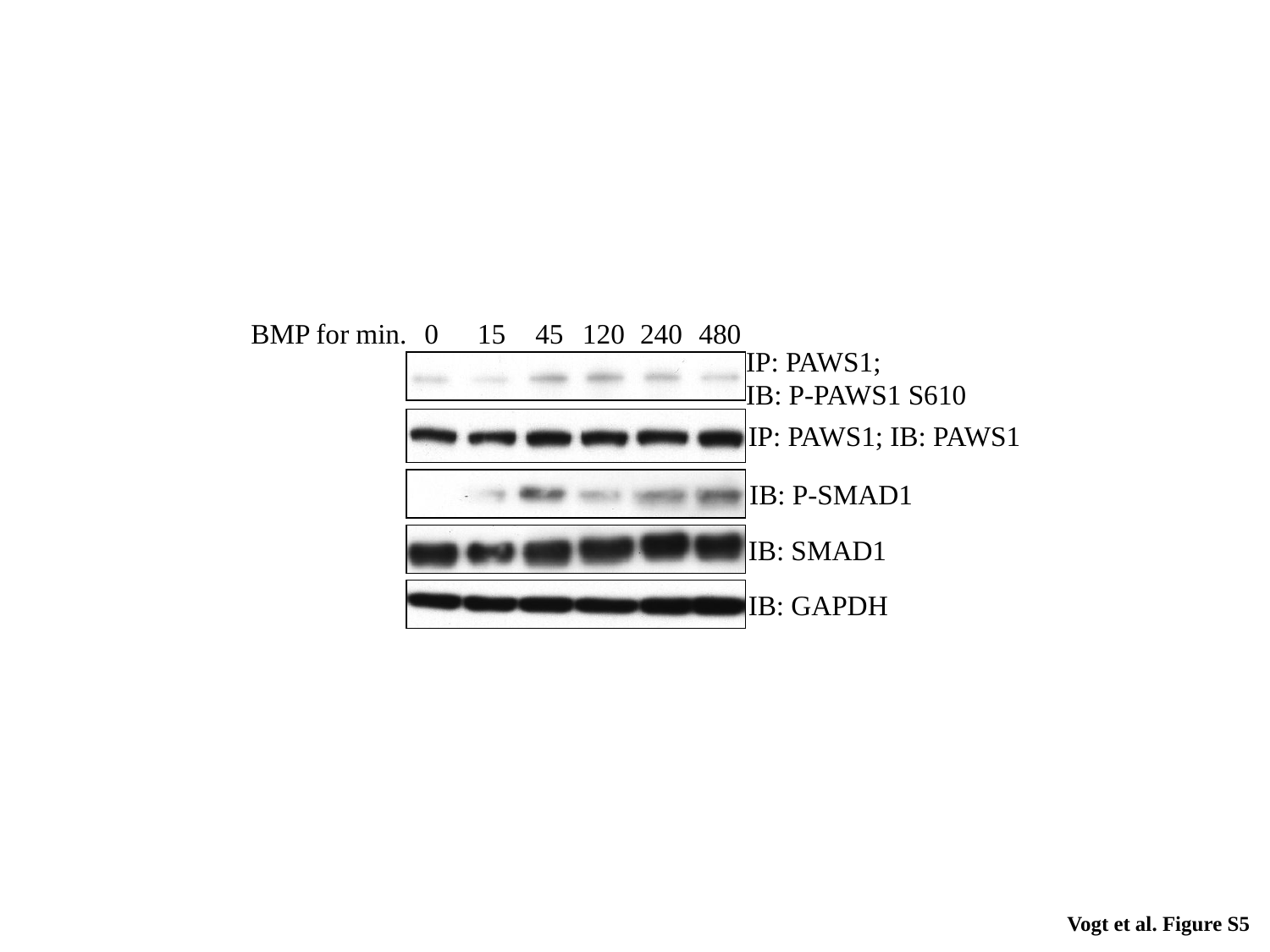

120
240
45
0
BMP for min.
15
480
IP: PAWS1;
IB: P-PAWS1 S610
IP: PAWS1; IB: PAWS1
IB: P-SMAD1
IB: SMAD1
IB: GAPDH
Vogt et al. Figure S5

## Slide 6
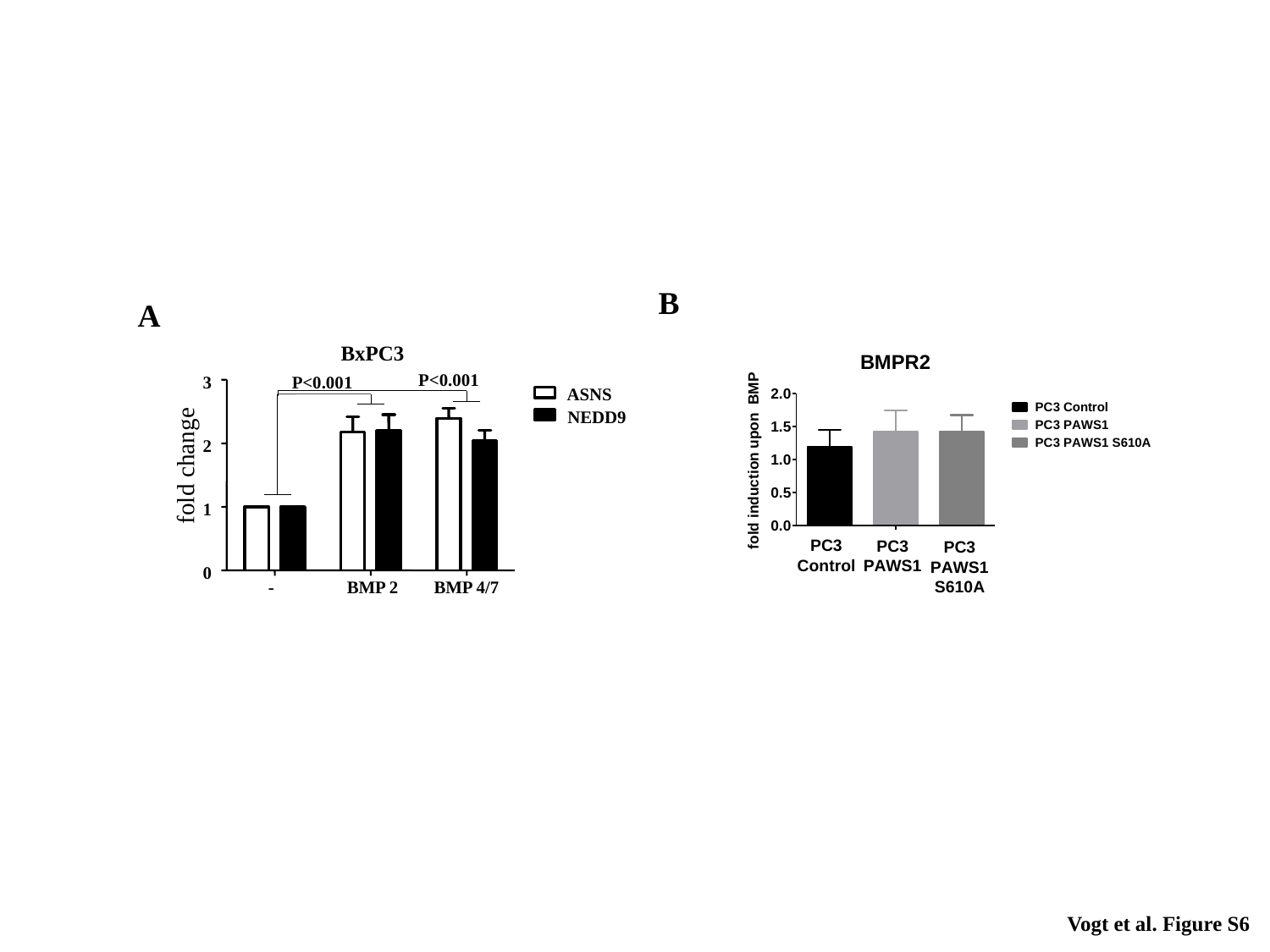

B
A
BxPC3
3
ASNS
NEDD9
2
fold change
1
0
-
BMP 2
BMP 4/7
P<0.001
P<0.001
Vogt et al. Figure S6

## Slide 7
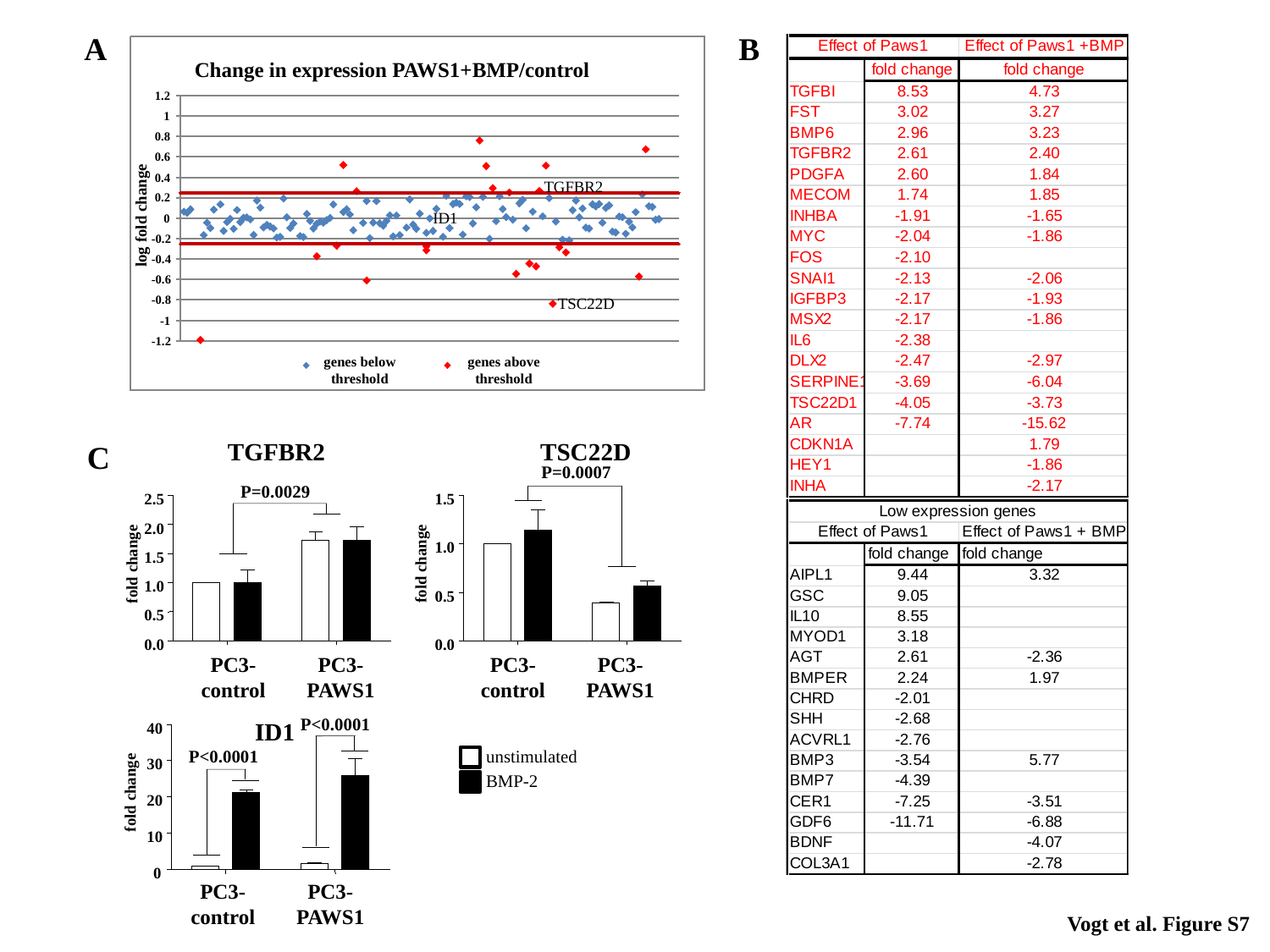

A
B
Change in expression PAWS1+BMP/control
1.2
1
0.8
0.6
0.4
0.2
log fold change
0
-0.2
-0.4
-0.6
-0.8
-1
-1.2
genes below threshold
genes above threshold
TGFBR2
ID1
TSC22D
C
TGFBR2
TSC22D
P=0.0007
P=0.0029
2.5
2.0
1.5
fold change
1.0
0.5
0.0
1.5
1.0
fold change
0.5
0.0
PC3-control
PC3-PAWS1
PC3-control
PC3-PAWS1
P<0.0001
ID1
40
30
fold change
20
10
0
PC3-control
PC3-PAWS1
P<0.0001
unstimulated
BMP-2
Vogt et al. Figure S7
